# A multicenter cross-sectional study in China revealing the intrinsic relationship between medical students’ grade and their perceptions of the learning environment

**DOI:** 10.1186/s12909-024-05538-4

**Published:** 2024-08-01

**Authors:** Runzhi Huang, Weijin Qian, Sujie Xie, Mei Cheng, Meiqiong Gong, Shuyuan Xian, Minghao Jin, Mengyi Zhang, Jieling Tang, Bingnan Lu, Yiting Yang, Zhenglin Liu, Mingyu Qu, Haonan Ma, Xinru Wu, Huabin Yin, Xiaonan Wang, Xin Liu, Yue Wang, Wenfang Chen, Min Lin, Chongyou Zhang, Erbin Du, Qing Lin, Zongqiang Huang, Jie Zhang, Guoyang Zhang, Yifan Liu, Yu Chen, Jun Liu, Shizhao Ji

**Affiliations:** 1 Department of Burn Surgery, the First Affiliated Hospital of Naval Medical University, No. 168 Changhai Road, Yangpu District, Shanghai, 200433 People’s Republic of China; 2 Research Unit of key techniques for treatment of burns and combined burns and trauma injury, Chinese Academy of Medical Sciences, 200433 Shanghai, People’s Republic of China; 3 Shanghai Jiao Tong University School of Medicine, Shanghai, 200025 China; 4 Department of Nephrology, the First Affiliated Hospital of Naval Medical University, 200433 Shanghai, People’s Republic of China; 5 Office of Educational Administration, Shanghai University, Shanghai, 200444 China; 6 Department of Orthopedics, Shanghai General Hospital, School of Medicine, Shanghai Jiaotong University, 100 Haining Road, Shanghai, China; 7 Department of Epidemiology and Health Statistics, School of Public Health, Capital Medical University, 10 Xitoutiao, Beijing, 100069 China; 8 Department of Rheumatology and Immunology, Second Affiliated Hospital of Naval Medical University, Shanghai, China; 9 Department of Health Statistics, School of Public Health, Air Force Medical University, No.169,Changle West Road, Xi’an, 710032 China; 10 Faculty of Medicine, Jinggangshan University, 28 Xueyuan Road, Ji’An, 343009 China; 11 Mental Health Education and Consultation Center,Chongqing Medical University, 61 Daxuecheng Middle Road, Chongqing, 401331 China; 12 Basic Medical College, Harbin Medical University, 157 Baojian Road, Harbin, 150081 Heilongjiang China; 13 Frist Clinical Medical College, Mudanjiang Medical University, 66 Tongxiang Street, Mudanjiang, 157011 China; 14 Department of Human Anatomy, Laboratory of Clinical Applied Anatomy, School of Basic Medical Sciences, Fujian Medical University, 1 Xuefu North Road, Fuzhou, 350122 China; 15 Department of Orthopedics, The First Affiliated Hospital of Zhengzhou University, 1 Jianshe East Road, Zhengzhou, 450052 China; 16 Department of Gynecology, Shanghai First Maternity and Infant Hospital, Tongji University School of Medicine, 2699 Gaoke West Road, Shanghai, 201204 China; 17 Maastricht University School of Health Professions Education, Maastricht, the Netherlands; 18 Department of Gyneacology and Obstetrics, The First Affiliated Hospital of Naval Medical University, Yangpu District, No. 168 Changhai Road, Yangpu District, Shanghai, 200433 China; 19 Department of Anesthesiology, Shanghai Pulmonary Hospital Affiliated to Tongji University, 507 Zheng Min Road, Shanghai, 200433 China

**Keywords:** Medical school learning environment, Johns Hopkins learning environment scale, Perception, Student’s grade, Nomogram, Intervention, Cross-sectional study

## Abstract

**Background:**

Medical school learning environment (MSLE) has a holistic impact on students’ psychosomatic health, academic achievements, and personal development. Students in different grades perceive MSLE in different ways. Thus, it is essential to investigate the specific role of student’s grade in the perception of MSLE.

**Methods:**

Using the Johns Hopkins Learning Environment Scale (JHLES) as a quantification instrument for the perception level of MSLE, 10,901 medical students in 12 universities in China were categorized into low or high JHLES group according to their questionnaires. We investigated the relationship between student’s grade and JHLES category by univariate analysis employing Pearson Chi-square test and Welch’s ANOVA. Then multivariable logistic regression analysis confirmed the predictive efficacy of student’s grade. A nomogram concerning the prediction of low JHLES score probability in medical students was also constructed.

**Results:**

A significant difference between two JHLES categories among students in different grades was observed (*p* < 0.001), with the proportion of the high JHLES group dominating in grade 1, 5, and the graduate subgroups (*p* < 0.001). The mean JHLES score declined especially in the third and fourth graders compared to freshmen (*p* < 0.001), while the mean score among the fifth graders had a remarkable rebound from the third graders (*p* < 0.001). Most imperatively, identified by multivariable logistic regression analysis, students in grade 3 (OR = 1.470, 95% CI = 1.265–1.709, *p* < 0.001) and 4 (OR = 1.578, 95% CI = 1.326–1.878, *p* < 0.001) perceived more negatively than freshmen. The constructed nomogram provided a promising prediction model for student’s low JHLES score probability, with accuracy, accordance, and discrimination (area under the curve (AUC) = 0.627).

**Conclusion:**

The student’s grade was a significant influencing factor in medical students’ perception of MSLE. The perceptions among the third and fourth graders got worse, probably due to the worrying changes in various aspects of MSLE during that period. The relevant and appropriate interventions to improve medical students’ perceptions are urgently needed.

**Supplementary Information:**

The online version contains supplementary material available at 10.1186/s12909-024-05538-4.

## Introduction

Learning environment (LE) is ordinarily defined as a psychosomatic and social learning climate provided by an academic institution where students experience the curriculum, the facilities, and the interactions with faculty and peers [1]. As one of the imperative elements in medical universities, medical school learning environment (MSLE) has been associated with students’ academic achievements, overall health, and contentment [2, 3]. A satisfying perception of a supportive MSLE can not only improve a student’s academic record, confidence, sense of wellbeing and personal development, but also diminish distress and self-doubt [3–8]. On the contrary, an unfavorable and stressful MSLE may give rise to burnout and apprehension [6, 9]. Seeking intrinsic or extrinsic influencing factors for MSLE and then promptly rectifying of undesirable factors are critical as well as indispensable for the formation of an advantageous MSLE, which can promote the students’ learning and the evolution of their professional identities [5].

The quality of a MSLE can be evaluated by medical students’ perceptions of it, and the latter can be quantitively measured by the Johns Hopkins Learning Environment Scale (JHLES) introduced by Shochet, R. B. et al. [5]. With 7 subscales and a total of 28 items, a higher JHLES score represents a more positive perception of MSLE. Conceivably, with the increment of a medical student’s grade, multifaceted variations take place simultaneously, which may result in changes of MSLE in diverse directions. For instance, from the matriculating students to the fourth graders, the curriculum and workload become increasingly burdensome, and most medical students will undergo a period of transition from a basic to a clinical learning style [10–13]. Previous studies have reported that medical students in different grades perceived MSLE differently, which has been confirmed in medical schools in a variety of countries, such as Sudan, Tunisia, Vietnam, and India [14–17]. For instance, a study has demonstrated an increase in the depression rate in senior medical students compared with freshmen, which might be related to the delicate changes in MSLE in part [18]. In addition, our precedent cross-sectional study involving medical students from 11 universities in China has elaborated that the student’s grade was one of the influencing factors for MSLE perception [19]. Despite previous efforts on the relationship between a student’s grade and MSLE, the concrete and exact impact of this potential influencing factor on student’s perception of MSLE has not been revealed. Therefore, it is essential to investigate the specific role of student’s grade in the perception of MSLE, so as to guide educators in developing appropriate and feasible interventions.

In this study, we mainly focused on elucidating the relationship between student’s grade and their MSLE perceptions. More precisely, we hoped to explore the discrepancy in MSLE perceptions among students in different grades along with the possible reasons, which might provide reliable evidence for specialists in the field of medical education to carry out corresponding interventions. The optimized MSLE will foster the shaping of students’ professional qualities as well as the development of the medical education, which is of great consequence.

## Materials and methods

### Sample selection and data extraction

This study (CHEC2023-284) was approved by the Ethics Committee of the First Affiliated Hospital of Naval Medical University. A multicenter, large-scale sample study was conducted in 12 universities in China, which included 6 university categories: the 985 Project Universities (Peking University and Tongji University), the 211 Project Universities (Zhengzhou University), the Non-985/211 Project Universities (Jinggangshan University), the First Batches of Medical Universities (Capital Medical University, Chongqing Medical University, Fujian Medical University, Harbin Medical University, and Southwest Medical University), the Second Batches of Medical Universities (Mudanjiang Medical University), and the military universities (Naval Medical University and Air Force Medical University). The medical students from different grades in these universities were selected to complete a questionnaire in which the subscales and items reflected their perceptions of MSLE.

Primarily, we performed a pilot study with 20 students in Naval Medical University selected by stratified random sampling according to their grade. Students from grade 1 to 5 and graduate students were coded on the basis of their student number and then integrated into the random number table for the corresponding grade. Subsequently, 3 undergraduate students in each grade and 5 graduate students were randomly selected to fill in the questionnaire. As the questionnaire was translated into Chinese according to the original template (see in 2.2), several items should be modified according to students feedbacks to improve its readability and rationality. We changed all questions into positive declarative sentences to better apply Likert scale (see later). We also changed some words for easier comprehension in Chinese, such as, “student” into “classmate”, “school” into “medical school”, “clinical” into “clinical field”, etc. A few possible predisposed terms were deleted or changed into neutral words. Ultimately, the accuracy as well as fluency of the questionnaire have been greatly improved. It was then integrated into a trustworthy and professional online platform called Wenjuanxing (https://www.wjx.cn/) for questionnaire inquiry.

Followingly, the link to the normative questionnaire was delivered to relevant responsible officers in medical schools of aforementioned 12 universities. Students were categorized by grade (from grade 1 to grade 5), and all registered students in 1 or 2 classes of each grade were randomly selected by stratified cluster sampling, who were included and then were invited to fill out the questionnaire. Graduate medical students from Tongji University, Capital Medical University, Chongqing Medical University, Fujian Medical University, Harbin Medical University, Mudanjiang Medical University, and Naval Medical University also participated in this study. Return students were excluded. Before distributing the questionnaire, all participated students were informed of the study’s purpose and the anonymity of their collaboration. 12,600 questionnaires were distributed to 12 universities and 11,265 of them were obtained from the respondents. Eventually, the unqualified questionnaires with inaccurate variables or unknown information were excluded, and a total of 10,901 valid questionnaires were downloaded for further analyses. The demographic information as well as other results were extracted and showed in a table integrally.

### JHLES instrument for the assessment of students’ perceptions of MSLE

The Johns Hopkins Learning Environment Scale (JHLES) was applied to evaluate the students’ perceptions of MSLE in this study. With great reliability, utility, and validity, JHLES comprises 7 subscales, including the community of peers with 6 items, the faculty relationships with 6 items, the academic climate with 5 items, the meaningful engagement with 4 items, the mentoring with 2 items, the inclusion and safety with 3 items, and the physical space with 2 items [5]. The detailed scale information was listed in Supplementary Table 1. The Likert scale method of five points ranging from strongly disagreed (1 point) to strongly agreed (5 points) was utilized to score each item, and the final score of 28 items altogether was calculated. A high JHLES score indicated a positive perception of MSLE, whereas a low score meant the opposite [5, 20].

### Statistical analysis

For the sake of exploring the probable influencing factors for perceptions of MSLE, the participants were classified into low or high JHLES score group by the median JHLES score (104) collected from this study. Pearson Chi-Square tests were performed to investigate the potential factors affecting the JHLES score, and the significant variables were displayed in a heatmap. Then, the student’s grade variable was extracted to excavate its relationship with the JHLES score, and a further Welch’s ANOVA analysis was conducted. Moreover, the proportion of two JHLES score groups and the mean JHLES score in different subgroups of the student’s grade were computed as well. The results were exhibited in bar, scatter, and violin plots.

In this study, we aimed to discover whether student’s grade was a possible predictor for JHLES score. We also incorporated significant factors in the nomogram to develop a multivariable prediction model in order to support decision making for the improvement of MSLE in the field of education. To substantiate that student’s grade was a potential predictor of students’ perceptions of MSLE, grade and 6 other demographic variables such as age, gender, ethnicity, major, native place, and grade point average (GPA) were integrated into the multivariable logistic regression analysis. The odds ratio (OR), 95% confidence interval (CI), and *p* value for each variable were demonstrated in a table. Additionally, a nomogram on the basis of the above 7 significant variables was constructed for better visualization and prediction of the low JHLES score probability for each participant. Tables displayed the score of each variable subclassification as well as the linear predictor in the nomogram. Furthermore, the efficaciousness and predictive accordance of the nomogram model was evaluated by C-index using bootstrap method, with 1000 iterations. The net benefits of the medical students were assessed by the decision curve analysis (DCA), while the calibration performance was evaluated by calibration curve, simultaneously. Afterwards, participants were randomized into a train set (70%) and a test set (30%) for internal validation with the application of “CreateDataPartition” function of the R package (version 4.1.3, www.r-project.org). The nomogram was established in the train set and validated in the test set, with receiver operation characteristic (ROC) curve validating the discrimination of nomograms in train, test and total sets. The outcomes were visualized respectively. Furthermore, the relationship between 28 items of JHLES and student’s grade was explored by Pearson Chi-Square test. Finally, a graphic abstract was created using Biorender website (https://biorender.com/).

In this study, the analyzing processes were performed in R (Institute for Statistics and Mathematics, version 4.1.3; www.r-project.org; Vienna, Austria) and SPSS20.0 (SPSS Inc., Chicago, IL, USA). For descriptive statistics, the continuous variables with normal distribution were described by mean ± standard deviation (SD), while the median with interquartile range was used for continuous variables with abnormal distribution. Number and percentage were utilized for categorical variables. Only a two-sided *p* value smaller than 0.05 was considered statistically significant.

## Results

### General information

**Fig. 1:**
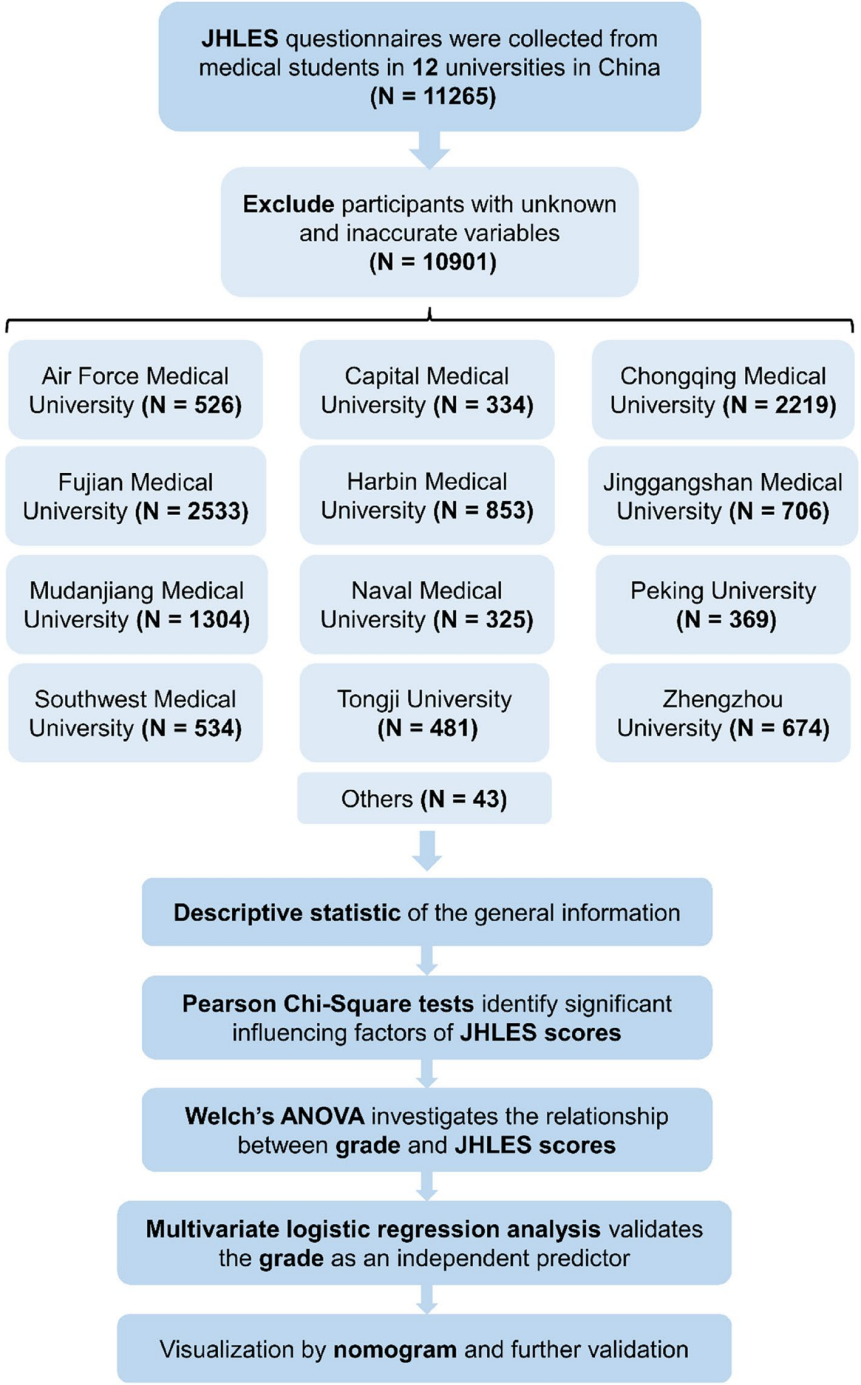
The flowchart for data extraction and subsequent analyses. JHLES, Johns Hopkins Learning Environment Scale

The flowchart of this study was displayed in Fig. 1. 12,600 questionnaires were distributed to the included medical students from 12 universities in China. Among them, 11,265 questionnaires in total were obtained, while only 10,901 valid questionnaires were selected for subsequent analyses. The respond and analysis proportion were 89.40% and 86.52%, respectively. The general information and characteristics of participants were showed in Table 1. The students’ ages ranged from 16 to 40 years, but the majority (98.09%) were between the ages of 16 and 25. Nearly 60% of all were female. Most students came from Fujian Medical University (23.24%), and a majority of students majored in clinical medicine (79.52%). In the aspect of student’s grade, almost 70% of students had a 5-year educational system, and there were more students in grade 1 comparatively with a percentage of 34.86, followed by grade 2 (18.74%), grade 4 (17.15%), grade 3 (15.28%), grade 5 (11.91%), and graduate students (2.06%). A large proportion of students’ parents had a low education level. Over 70% of students regarded their learning environment in schools and doctor-patient relationship in hospitals as good or excellent, and only a fraction of students was uninterested in medicine (2.11%). According to the JHLES category, 54.46 percent of students thought their MSLE was good.

**Table 1:** Baseline Characteristics of 10,901 participants

**Variables**	Number (Percentage)
**Age**	
16–20	5868 (53.83)
21–25	4825 (44.26)
26–40	208 (1.91)
**Gender**	
Female	6531 (59.91)
Male	4370 (40.09)
**University category**	
211 Project Universities	692 (6.35)
985 Project Universities	853 (7.82)
Military University	851 (7.81)
Non_985_211 Project Universities	720 (6.60)
The First Batches of Medical Universities	6473 (59.38)
The Second Batches of Medical Universities	1312 (12.04)
**University**	
Air Force Medical University	526 (4.83)
Capital Medical University	334 (3.06)
Chongqing Medical University	2219 (20.36)
Fujian Medical University	2533 (23.24)
Harbin Medical University	853 (7.82)
Jinggangshan University	706 (6.48)
Mudanjiang Medical College	1304 (11.96)
Naval Medical University	325 (2.98)
Peking University	369 (3.39)
Southwest Medical University	534 (4.90)
Tongji University	481 (4.41)
Zhengzhou University	674 (6.18)
Others	43 (0.39)
**Major**	
Clinical medicine	8668 (79.52)
Nursing	572 (5.25)
Phylaxiology	698 (6.40)
Preclinical medicine	658 (6.04)
Stomatology	305 (2.80)
**Ethnicity**	
Ethnic Han	10,190 (93.48)
Minority	711 (6.52)
**Only child**	
No	6140 (56.33)
Yes	4761 (43.67)
**Grade**	
Grade 1	3800 (34.86)
Grade 2	2043 (18.74)
Grade 3	1666 (15.28)
Grade 4	1869 (17.15)
Grade 5	1298 (11.91)
Graduate	225 (2.06)
**Native place**	
Country	2562 (23.50)
Municipality	1535 (14.08)
Prefecture city	2063 (18.92)
Provincial capital	1127 (10.34)
Town	1196 (10.97)
Village	2418 (22.18)
**Educational system**	
Eight-year	1305 (11.97)
Seven-year	280 (2.57)
Five-year	7621 (69.91)
Other	1695 (15.55)
**GPA**	
Top 5%	815 (7.48)
5–20%	2509 (23.02)
20–50%	3844 (35.26)
50–80%	2687 (24.65)
80–100%	1046 (9.60)
**Father’s education level**	
Graduate degree	251 (2.30)
Bachelor degree	1292 (11.85)
Junior college	1141 (10.47)
Senior high school	2623 (24.06)
Junior high school	3800 (34.86)
Preliminary school	1794 (16.46)
**Father’s occupation**	
Civil servant	1083 (9.93)
Company employee	1093 (10.03)
Freelance work	2112 (19.37)
Individual household	1092 (10.02)
Professional/technical	1150 (10.55)
Worker/peasant	4371 (40.10)
**Mother’s education level**	
Graduate degree	174 (1.60)
Bachelor degree	959 (8.80)
Junior college	1017 (9.33)
Senior high school	2249 (20.63)
Junior high school	3322 (30.47)
Preliminary school	3180 (29.17)
**Mother’s occupation**	
Civil servant	634 (5.82)
Company employee	1250 (11.47)
Freelance work	2892 (26.53)
Individual household	791 (7.26)
Professional/technical	1363 (12.50)
Worker/peasant	3971 (36.43)
**Learning environment of your schools**	
Terrible	63 (0.58)
Bad	125 (1.15)
Common	2284 (20.95)
Good	6048 (55.48)
Excellent	2381 (21.84)
**Doctor-patient relationship in your hospitals**	
Terrible	46 (0.42)
Bad	121 (1.11)
Common	2812 (25.80)
Good	6190 (56.78)
Excellent	1732 (15.89)
**Interests of Medicine**	
Extremely uninterested	65 (0.60)
Uninterested	165 (1.51)
Common	2654 (24.35)
Interested	6145 (56.37)
Extremely interested	1872 (17.17)
**JHLES category**	
High (> = 104)	5937 (54.46)
Low (< 104)	4964 (45.54)

*GPA* grade point average, *JHLES* Johns Hopkins Learning Environment Scale

### Identify influencing factors with statistical significance of JHLES score

**Fig. 2:**
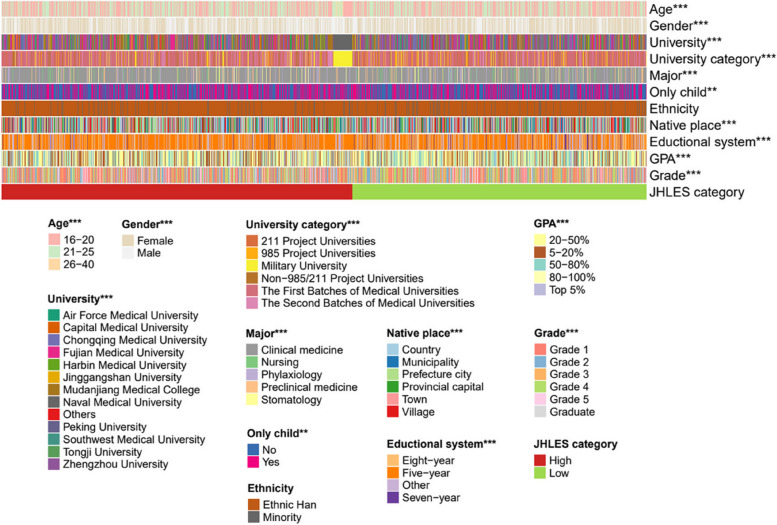
Pearson Chi-Square tests identify the influencing factors with significant differences between the high and low JHLES categories. GPA, grade point average; JHLES, Johns Hopkins Learning Environment Scale

Ten thousand nine hundred one students were divided into high or low JHLES category according to the median score (104), where a high or low score was consistent with positive or negative perceptions towards MSLE. Preliminarily, the Pearson Chi-Square test was implemented to identify the striking influencing factors for JHLES score, and 10 significant variables (age, gender, university, university category, major, only child, native place, educational system, GPA, and grade) as well as a demographic variable, ethnicity, were holistically demonstrated in Fig. 2. The “***” and “**” at the upper right corner of each variable represented its *p* value < 0.001 or 0.01, respectively. Whereafter, student’s grade variable with great significance was chosen to study its impact on the JHLES score.

**Fig. 3:**
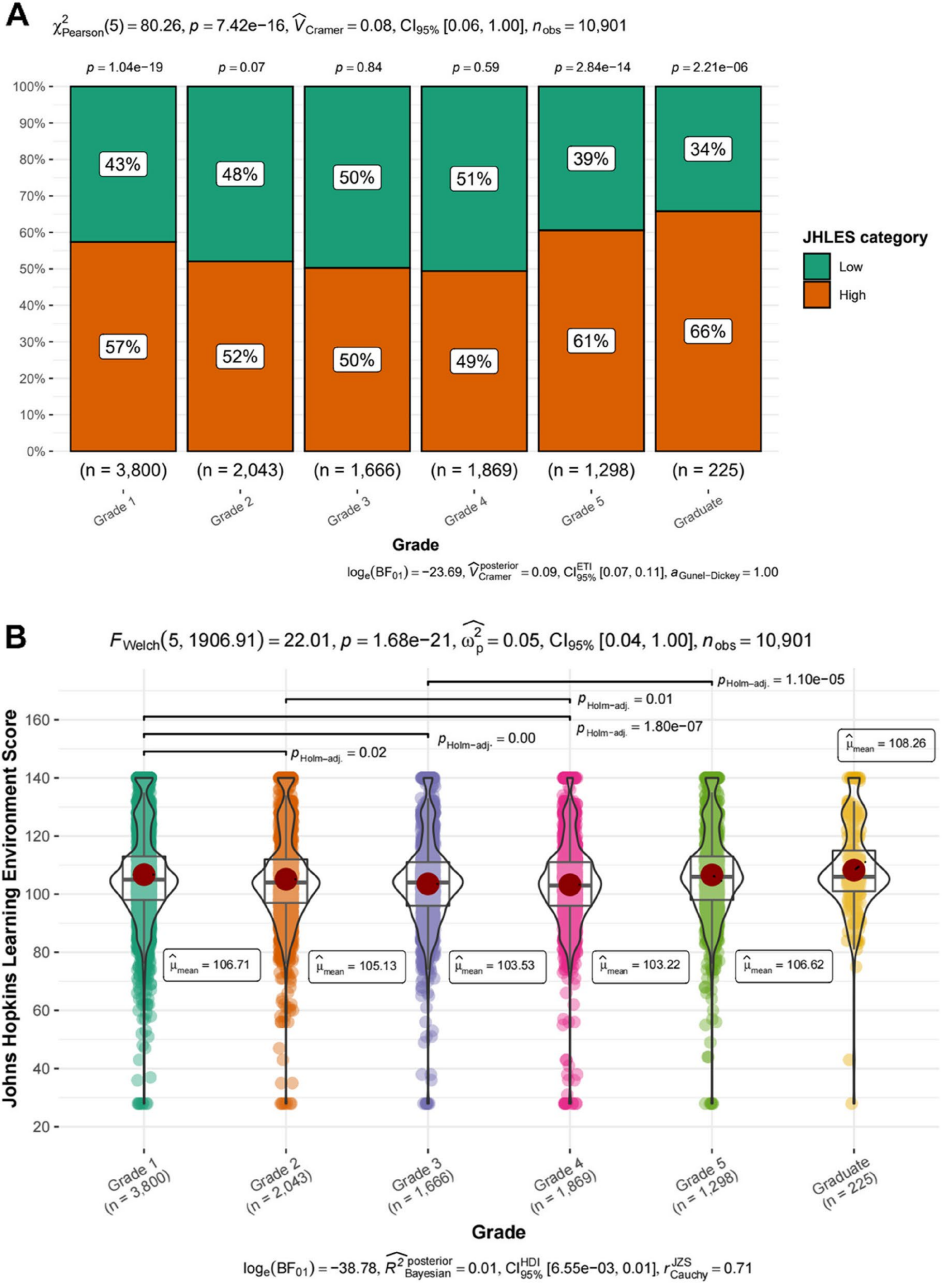
The Pearson Chi-Square test and Welch’s ANOVA explore the differences in the JHLES score among students from different grades. **A** Pearson Chi-Square test showed significant differences in the JHLES score in terms of the student’s grade by bar plots (*p* < 0.001). **B** Significant differences in the mean JHLES score of students from different grades were suggested by Welch’s ANOVA and visualized in scatter plots and violin plots (*p* < 0.001). JHLES, Johns Hopkins Learning Environment Scale

Figure 3A revealed the notable difference between two JHLES categories among students in different grades (*p* < 0.001). Furthermore, there was a significant difference in the composition of the high and low JHLES categories in grade 1, grade 5, and graduate students with *p* values less than 0.001, with the high JHLES category clearly dominating. It suggested that the medical students’ perceptions of MSLE were more positive and energetic among freshmen, grade 5 and the graduate stage. Despite the fact that the proportion of students in low JHLES category elevated stepwise from grade 2 to grade 4, eventually surpassing the high group (49%) in grade 4, there were no significance of JHLES score in these 3 grades (*p* > 0.05).

Subsequently, Welch’s ANOVA indicated the significant differences (*p* < 0.001) in the mean JHLES score of students from different grades (Fig. 3B). From grade 1 to grade 4, the mean JHLES score gradually declined from 106.71 to 103.22, especially in the third and fourth graders compared to freshmen (*p* < 0.001), which denoted the students’ perceptions of MSLE getting worse in grade 3 and 4. The mean score of the fourth graders was also significantly below that of the sophomores (*p* < 0.05). However, the mean JHLES score among the fifth graders (106.62) was strikingly higher compared to the third (*p* < 0.001), and the graduates’ mean score was even higher (108.26). Conceivably, the results were in accordance with those of the Pearson Chi-Square tests above. More positive perceptions of MSLE were observed in freshmen, sophomores, fifth graders, and graduates rather than in third and fourth graders. Moreover, the individual difference was also an important issue as the JHLES score ranged from the minimum 28 to the maximum 140 within a single grade. As the external environment was similar in the same grade, the own characteristics and personal factors of students may contribute to the discrepancy of their perceptions towards MSLE.

### Multivariable logistic regression analysis for validating student’s grade as a potential predictor

To ulteriorly explore whether the student’s grade was a potential predictor for the medical students’ perceptions of MSLE, the grade together with 6 other demographic variables (age, gender, ethnicity, major, native place, and GPA) were incorporated into the multivariable logistic regression analysis. The statistics data like OR, 95% CI and *p* value were depicted in Table 2. It was discovered that the age of 26–40 and male were both positively related to high JHLES score probability and further, the better perception of MSLE (OR = 0.426, 95% CI = 0.283–0.642, *p* < 0.001; OR = 0.733, 95% CI = 0.675–0.795, *p* < 0.001). Next, students who majored in nursing or phylaxiology were more likely associated with worse perceptions (OR = 1.494, 95% CI = 1.248–1.788, *p* < 0.001; OR = 1.272, 95% CI = 1.085–1.491, *p* = 0.003), yet the native places of students such as the prefecture city and provincial capital were considered to have a converse effect on students’ perceptions (OR = 0.790, 95% CI = 0.701–0.891, *p* < 0.001; OR = 0.762, 95% CI = 0.659–0.881, *p* < 0.001). Conspicuously, GPA in all subgroup levels was significant with a *p* value < 0.001. Higher GPA levels of students were associated with more satisfying and positive perceptions of MSLE.

**Table 2:** Multivariable logistic regression analysis of JHLES scores

Variables	JHLES score	
	**OR (95% CI)**	***P* value**
**Age**		
16–20	1.000 (Reference)	
21–25	0.926 (0.806–1.063)	0.275
26–40	0.426 (0.283–0.642)	< 0.001*
**Gender**		
Female	1.000 (Reference)	
Male	0.733 (0.675–0.795)	< 0.001*
**Ethnicity**		
Ethnic Han	1.000 (Reference)	
Minority	0.880 (0.752–1.030)	0.110
**Major**		
Clinical medicine	1.000 (Reference)	
Nursing	1.494 (1.248–1.788)	< 0.001*
Phylaxiology	1.272 (1.085–1.491)	0.003*
Preclinical medicine	1.046 (0.888–1.232)	0.590
Stomatology	0.801 (0.630–1.020)	0.071
**Native place**		
Country	1.000 (Reference)	
Municipality	1.050 (0.921–1.197)	0.462
Prefecture city	0.790 (0.701–0.891)	< 0.001*
Provincial capital	0.762 (0.659–0.881)	< 0.001*
Town	0.916 (0.795–1.054)	0.220
Village	0.956 (0.853–1.072)	0.443
**GPA**		
20–50%	1.000 (Reference)	
5–20%	0.763 (0.687–0.847)	< 0.001*
50–80%	1.484 (1.342–1.641)	< 0.001*
80–100%	1.871 (1.624–2.156)	< 0.001*
Top 5%	0.522 (0.443–0.617)	< 0.001*
**Grade**		
Grade 1	1.000 (Reference)	
Grade 2	1.242 (1.109–1.391)	< 0.001*
Grade 3	1.470 (1.265–1.709)	< 0.001*
Grade 4	1.578 (1.326–1.878)	< 0.001*
Grade 5	1.067 (0.883–1.290)	0.500
Graduate	0.830 (0.570–1.208)	0.330

*JHLES* Johns Hopkins Learning Environment Scale, *OR* odds ratio, *CI* confidence interval, *GPA* grade point average

^*^P < 0.05

Finally, employing the grade 1 subgroup as a reference, the grade 2 (OR = 1.242, 95% CI = 1.109–1.391, *p* < 0.001), grade 3 (OR = 1.470, 95% CI = 1.265–1.709, *p* < 0.001), and grade 4 (OR = 1.578, 95% CI = 1.326–1.878, *p* < 0.001) were significant risk factors for low JHLES probability, which indicated that students in these 3 grades perceived worse of MSLE than the freshmen group. Nevertheless, the fifth graders and the graduates born no significance from students in grade 1. In conclusion, after the integration of demographic data in multivariable logistic regression analysis the student’s grade still remained a potential predictor for medical students’ perceptions of MSLE.

### A nomogram for prediction of low JHLES score probability and further validation

**Fig. 4:**
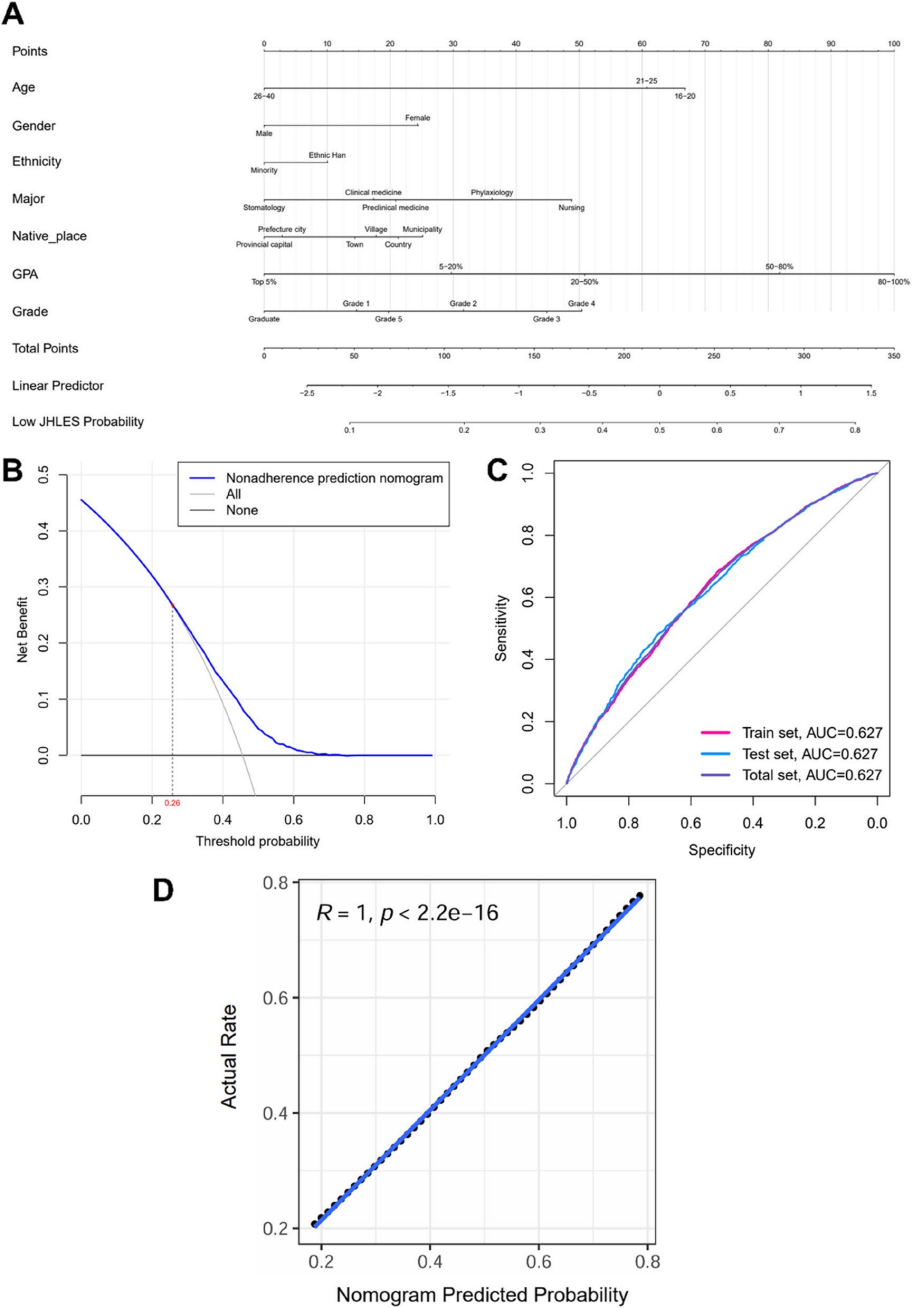
Construction and validation of the nomogram. **A** A nomogram for predicting the low JHLES score probability of medical students. **B** The DCA of the nomogram. The X axis represented the range of threshold probability based on the nomogram, at which an intervention might be applied. The Y axis typically represented the net benefit, which was calculated by weighing both the benefits (true positives) and harms (false positives) associated with intervention across different threshold probability. The curve’s shape and position relative to reference lines (treating everyone or treating no one) help assess the clinical usefulness of the nomogram. When the low JHLES probability of the nonadherence prediction nomogram reached 0.26, giving proper interventions to students could obtain higher net benefits compared to treating every student. **C** The ROC curves of the nomogram for the train, test, and total sets (AUC = 0.627), indicating excellent predictive accordance and discrimination. **D** The calibration curve for the nomogram. The X axis represents the nomogram-predicted probability, and Y axis represents the actual probability of a low JHLES score. The fitted curve (blue line, R = 1) of the black dots indicates great performance of the nomogram. JHLES, Johns Hopkins Learning Environment Scale; DCA, decision curve analysis; ROC, receiver operating characteristic; AUC, area under the curve

A nomogram containing the aforementioned 7 variables was constructed for JHLES score prediction as well as visualization (Fig. 4A). The subgroups of each variable were assigned corresponding points in the nomogram based on the multivariable logistic regression analysis, listed in Table 3. It was worth noting that a student with higher total points in the nomogram model would be more likely to possess a lower JHLES score, representing his or her poorer perception of MSLE. In the nomogram, the score for age 16–20 (67) was higher than 21–25 (61) and 26–40 (0). Females as well as ethnic Han were inclined to have a low JHLES score. In terms of major, nursing students might have the worst perceptions with 49 points, followed by students of phylaxiology (36), preclinical medicine (21), clinical medicine (17), and stomatology (0). Moreover, students from the provincial capital (0) and prefecture city (3) tended to gain a favorable perception of MSLE in contrast with students from the municipality (25), country (21), village (18), and town (14). For GPA, a higher GPA was always correlated with better perception. Most imperatively, there was not a one-way trend of increasing or decreasing points across different students’ grades, which was of great interest. The third and fourth graders scored higher (45 and 50, respectively), while freshmen (15), sophomores (32), the fifth graders (20), and the graduates (0) held lower points, which strongly indicated that students in grade 3 and 4 perceived MSLE as being more inferior, discouraging and unfavorable in comparison with students in grade 1, 2, 5, and the graduates. The prediction of the nomogram’s total points for low JHLES probability was particularized in Table 4.

**Table 3:** Scores of variables in the nomograms

Variables	Points
**Age**	
16–20	67
21–25	61
26–40	0
**Gender**	
Female	24
Male	0
**Ethnicity**	
Ethnic Han	10
Minority	0
**Major**	
Nursing	49
Phylaxiology	36
Preclinical medicine	21
Clinical medicine	17
Stomatology	0
**Native place**	
Municipality	25
Country	21
Village	18
Town	14
Prefecture city	3
Provincial capital	0
**GPA**	
80–100%	100
50–80%	82
20–50%	51
5–20%	30
Top 5%	0
**Grade**	
Grade 1	15
Grade 2	32
Grade 3	45
Grade 4	50
Grade 5	20
Graduate	0

*GPA* grade point average

**Table 4:** Total points in the nomogram predicted the probability of low JHLES score

Total Points	Low JHLES Probability
328	0.8
286	0.7
252	0.6
220	0.5
188	0.4
153	0.3
111	0.2
48	0.1
Points per unit of linear predictor	78.373
Linear predictor units per point	0.0127595

*JHLES* Johns Hopkins Learning Environment Scale

The DCA curve unearthed that when the low JHLES probability of the nonadherence prediction nomogram reached 0.26, giving proper interventions to students could obtain higher net benefits compared with treating every student (Fig. 4B). If the probability predicted by the nomogram was lower than the threshold value, it was better not to intervene in order to avoid overcorrection. Besides, the efficacy of the nomogram was also evaluated by the ROC curve, of which all three area under the curves (AUCs) in the train, test, and total sets were 0.627 (Fig. 4C), suggesting a modest predictive accordance, accuracy, and discrimination. The calibration curve in Fig. 4D displayed the nomogram’s great performance and consistency with the physical truth.

### Further study on the relationship between student’s grade and subscales of JHLES

A descriptive heatmap clustered by student’s grade was generated in order to intuitively exhibit the distribution of 28 items’ score of JHLES for each student (Figure S1). Subsequently, Pearson Chi-Square test was performed for students in grade 3 and 4 as well as other graders, showing that all 28 items scores were significantly different between the two grade categories (*p* < 0.001, Figure S2), which indicated MSLE changes in various aspects between grade 3/4 and other grades.

**Fig. 5:**
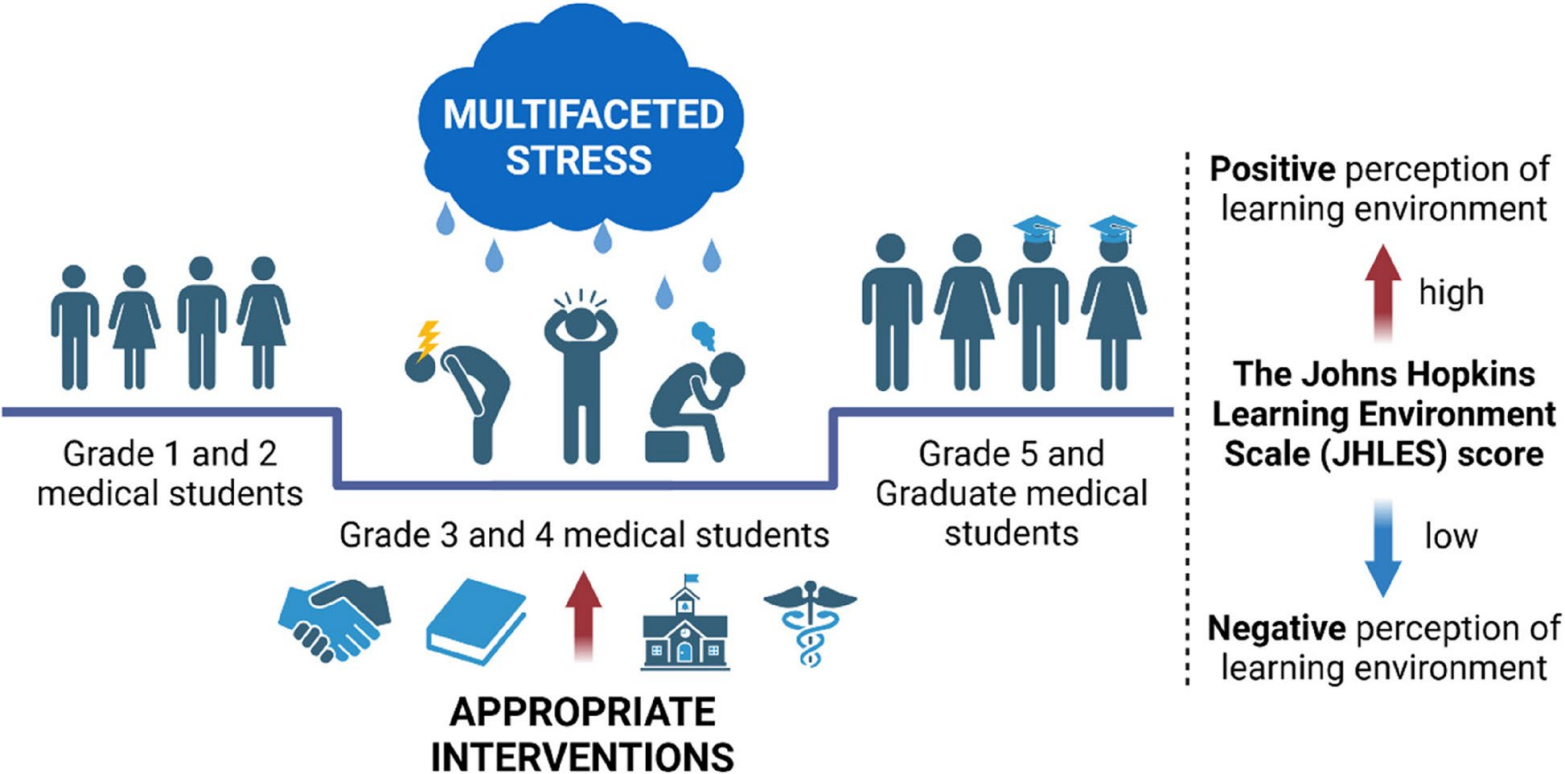
The summary graph of the relationship between students’ grades and their perceptions of MSLE. MSLE, medical school learning environment

After meticulously discussing the reasons for the negative perception of MSLE among students in grade 3 and 4, together with the corresponding interventions, a graphic abstract was generated, showed in Fig. 5.

## Discussion

In this study, 10,901 responses of the JHLES questionnaire from students in 12 medical universities in China were collected. After employing Pearson Chi-Square tests and Welch’s ANOVA, students’ grade was ascertained as a significant influencing factor, and a lower mean JHLES score was observed in grade 3 and 4 subgroups compared to grade 1 (or grade 2). Whereafter, student’s grade was validated as a potential predictor of MSLE perceptions in multivariable logistic regression analysis. Interestingly, the third and fourth graders were more likely to perceive MSLE negatively than matriculating students, while there was no significance for the fifth graders or the graduates. Subsequently, a nomogram was generated to better predict the probability of low JHLES score, that is, the probability of poor MSLE perception, showing modest predictive performance.

This study has concluded that, in terms of medical student’s perception of MSLE, the student’s grade was a potential predictor. During the first four years of study, their perceptions became more negative annually. The matriculating students’ perceptions were strikingly better than those of students in grade 3 and 4, while the fifth graders and the graduates perceived MSLE similarly to freshmen. However, the diagnostic tools for the nomogram exhibited contradictory results. The calibration curve showed excellent accordance with actual situation of the nomogram, while the AUC value of ROC curve only reached 0.627, indicating a not that good predictive accuracy. As stated in a previous article, the ROC performance may be fluctuated according to different clinical situations, like participant populations [21]. In our study, 10,901 students were included to construct the predictive nomogram. The large sample size might affect the ROC performance. Although the AUC value was low, it at least represented the association between student’s grade and JHLES score, which was supported by a large number of literatures. Anterior studies have reached a semblable conclusion, suggesting our results were of great reliability and uniformity. For instance, students majoring in occupational therapy have scented out a reduction of teaching quality from grade 1 to 3, which was a component of MSLE [22]. Using the Dundee Ready Educational Environment Measure (DREEM) questionnaire, it was discovered that the preclinical students (grade 1 and 2) perceived a more favorable LE than the clinical students (grade 3 and 4) in both India and Vietnam medical schools [16, 23, 24]. Meanwhile, the fifth- and sixth-year medical students in Thailand possessed better academic and social perceptions than the fourth graders, and the graduates or residents perceived the education environment optimistically [25–27]. Conservatively speaking, ROC curve was a necessary procedure for the diagnosis of model efficiency, and it implied a modest predictive performance of our nomogram. Therefore, the interpretation as well as application of the nomogram should be cautious. Additionally, the practical situation ought to be taken into account. Further studies with higher level of evidence such as prospective study, intervention study, or randomized controlled trial (RCT) are recommended in order to improve the study’s accuracy and clinical transformation efficacy.

The evident deterioration of MSLE perceptions of students in grade 3 and 4 might be correlated with the changes in multiple facets of MSLE along with the accompanying stress. Firstly, due to the curriculum provision system for medical students in China, the third and fourth graders were exposed to clinical courses and were requested to enter the hospital for probation. The quantified MSLE perception score was found to substantially decrease at the stage of preclinical-clinical transition [10, 11]. In this lengthy transition from the classroom-based LE to the clinical-based LE, students were required to face a completely novel teaching method, which was quite tough. Besides, some students would be apprehensive about their insufficient knowledge during the probationary period, with a sense of incompetence and unpreparedness [28]. Also, they were worried about making mistakes and the lack of an inclusive atmosphere in the clinical LE [29, 30]. Meanwhile, in addition to the perceptual changes, physical space was also changed at preclinical-clinical transition period. Before grade 3, students were often arranged to study in medical universities with comprehensive and well-equipped facilities, such as library, canteen, and gymnasium, which could meet students’ daily needs. After they entered the clinical stage, students may be admitted to the dormitory provided by hospitals with less sense of university. Besides, negative factors like conflicts between patients and doctors would worsen the clinical learning environment, leading to a more passive perception of MSLE by third and fourth graders [31].

Secondly, the third and fourth graders on probation had fewer opportunities to interact with their classmates, compared to their junior freshmen and sophomores who learned altogether in a single classroom by class [10]. An earlier study has illustrated that freshmen and sophomores involved in the learning community (LC) were apt to get more help from their companions, which led to a positive perception of MSLE [32]. As for students in grade 3 and 4, they may gradually realize the responsibility and duty of becoming medical workers, and would spend more time on their heavy schoolwork instead of attending social activities and interacting with peers. Thus, the supportive community of peers and the mutual assistance obtained from each other were attenuated among students in grade 3 and 4, resulting in their much more anxious perceptions of MSLE [15].

Thirdly, unlike other majors, medicine is a subject that a person needs to devote himself to continuously studying throughout his life. A wide range of courses, including basic courses like immunology, genetics, and cell biology, together with clinical courses such as internal medicine and surgery, were allocated in the first four years’ schedule for medical students. With the increase in a student’s grade, the relative curriculum became more onerous. The gradual increase in academic and examination pressure reached a peak in the fourth year of study, which was discouraging for the fourth graders. Some students at this period may suffer from the tight curriculum schedule and frequent test arrangement, which contributed to a not so good academic climate. It could partially explain their poor perceptions of MSLE [13, 15].

Fourthly, some universities in China have advocated the introduction of scientific research into MSLE of undergraduates since the third year of study. Despite the fact that this integration fostered students’ subsequent academic achievements and cultivated their research skills as well as critical thinking, it has brought the third and fourth graders a certain amount of pressure [33, 34]. For instance, students may find it challenging to match a suitable mentor in their interested specialty. Certain fields of research and clinical practice may face a scarcity of mentors with sufficient expertise, while in popular or highly competitive field, students would need to compete with other interested peers to acquire the favor of their dreamed mentors. Also, mentors sometimes were too busy to pay close attention to students due to heavy workload and numerous research projects, leading to disappointment and unmet expectations. Although being mentored is deemed to have a positive influence on career development of students, the above difficulties hindered students’ improvement [35].

Last but not least, medical students in grade 3 and 4, especially grade 4, were additionally confronted with the pressure of applying for a higher degree through the postgraduate entrance exam or the exemption of the admission exam. Because of the cultivating pattern of Chinese medical students, a majority of graduates with a bachelor’s degree desired to enter a higher learning institution for the purpose of learning more professional knowledge. Therefore, they were supposed to accumulate more stress in this process to a certain extent. In conclusion, based on the five reasons listed above that increase anxiety, the perceptions of MSLE among students in grade 3 and 4 were indeed worse than those among freshmen and sophomores.

As for the fifth graders, there was a partial recovery of the perception score of MSLE. During their internships, the previous pressure from schoolwork, examinations, and the application for a higher degree suddenly disappeared. Students often felt untrammeled to strive for the knowledge they yearned for [36]. They also gained abundant experience through the teaching and practice of physicians and their interactions with patients in the clinical LE [36]. Furthermore, they could exchange information and related resources with peers to help each other navigate the stressful preclinical-clinical transition period [37], creating a supportive climate that might be beneficial to promote the development of their MSLE perceptions in a positive direction. For the graduates, they paid more attention to their professional responsibilities after accessing a new stage of study, which might change their primary perceptions of MSLE [38].

A small number of studies have drawn opposite conclusions from our study. In two medical schools in Pakistan and Turkey, medical students in grade 3 and 4 seemed to perceived better than freshmen, according to the DREEM questionnaire [39, 40]. The existing discrepancy was presumably due to different students’ adaptations to clinical tasks, different teaching method or medical curricula in different countries. In addition, students’ perception of MSLE might be influenced by surrounded social relationships with fellow students, faculty and other members in the university, which could be discrepant across various culture settings. It’s of great importance to conduct further research on these influencing factors to gain a more complete understanding of the MSLE perceptions by medical students in different grades.

Apart from the discrepancy of student’s perception of MSLE among 6 grades, there were also difference within each grade, which could be partly attributed to individual factors of students. As displayed in Pearson Chi-Square test, GPA and whether being an only child were significant factors for JHLES score. Previous studies have concluded that a good perception of MSLE contributed to better academic performance [41, 42], while better academic performance, measured by GPA in most of medical universities in China, would provide students with sense of achievement together with more learning engagement for a higher goal, which could indeed promote their positive perception of MSLE. Moreover, being an only child was usually related to more concern and solicitude shone from parents as well as a more supportive atmosphere around, which may alleviate student’s daily pressure and improve their positive perception. Besides, the variance of MSLE perception may also due to distinct personality traits of students, such as extroverted, cooperated, or introverted character, which would affect their perception and engagement model in MSLE. Nonetheless, studies in terms of the influence of student’s personal factors on MSLE perception were still a scarcity, and further research is in an urgent need.

To promote academic performance, psychosomatic health, and the development of students at all grades, it is necessary for the professionals in the education field to take related interventions into effect to improve MSLE for medical students in grade 3 and 4 [29]. A peer teaching program has exhibited its remarkable efficacy in promoting communication between students in higher and lower grades or within peers [32, 34, 43], which proved to ease the fretfulness of the preclinical-clinical transition for junior graders and also elevate the teaching skills and the comprehension of medical knowledge for senior graders. What’s more, students-paired clinical placements have exerted a favorable effect on their MSLE and health [44]. As another intervention, adjusting the course structure, such as through the application of the integrated curriculum, could enhance students’ academic performance along with their perceptions of MSLE [45, 46]. Besides, a supportive educational environment was recommended, encompassing an encouraging teacher-student relationship, timely feedback and close attention from teachers, which profoundly contributed to students’ satisfaction with MSLE, as well as alleviated the stress of the transition period [47–49]. Additionally, the simulation-based training for students on probation was another intervention to benefit their transition to clinical placements [50]. More feasible interventions are expected to be formulated in the near future to advance MSLE and students’ developmental potential. The graphic abstract of this study was presented in Fig. 5.

Limitations still exist in this cross-sectional study. First, as it was defined as a cross-sectional study, only correlation can be provided rather than causal inference. It was unable to determine whether student’s grade or some other underlying factors caused the variation in the perception of MSLE. Second, even though the five-point Likert scale from strongly disagree to strongly agree was expounded in the questionnaire, some participants may respond to the items subjectively, which might influence the veracity and credibility of the collected data (reporting bias). Third, although a structural stratified random sampling method was utilized for participants selection, there still existed some systematic error. Moreover, as 6 grades were designed for pairwise comparisons, a total of 15 times comparisons increased the probability of a significant result, which was called the alpha error. Cross validation is recommended to verify whether the associations observed on our data are maintained on the new data. Fourth, despite the 7 subscale scores of JHLES were collected along with the total score, we just displayed and discussed the result instead of exploring thoroughly which aspect of MSLE could be deeply influenced by grade. Last but not least, the stressors and appropriate interventions for students with negative perceptions of MSLE pointed out in this article should be investigated in future studies, which was of great significance for medical educators and decision makers. Nevertheless, our study is still the first to concentrate on the intrinsic relationship between a student’s grade and a student’s perception of MSLE in China, in which the multi-center design and a huge sample size guarantee representative and typical results. Further research with higher level of evidence such as case–control study, cohort study and so on is urgently needed, in order to investigate the causal relationships between a medical student’s perception of MSLE and grade or other factors such as the GPA, major, and university category.

## Conclusion

The medical student’s grade was validated as a potential predictor for the medical student’s perception of MSLE, and the nomogram was also constructed as a reliable tool for the prediction of the student’s negative MSLE perception. The third and fourth graders held a higher probability of perceiving MSLE negatively probably due to the preclinical-clinical transition in MSLE, less support from peers, heavier workload, emerging research pressure, and the stress of applying for a higher degree. Consequently, appropriate interventions are urgently needed to improve the quality of MSLE among students in grade 3 and 4.

### Supplementary Information

Supplementary Material 1.

## Data Availability

The datasets generated and/or analyzed during the current study are available in the supplementary material.
