# Supplementary material for: A multicenter cross-sectional study in China revealing the intrinsic relationship between medical students’ grade and their perceptions of the learning environment
Source: BMC Med Educ. 2024 Aug 1;24:832. doi: 10.1186/s12909-024-05538-4 (PMC11295695; doi:10.1186/s12909-024-05538-4)
Supplement: Supplementary file 1 — Supplementary Material 1. [file 12909_2024_5538_MOESM1_ESM.docx]

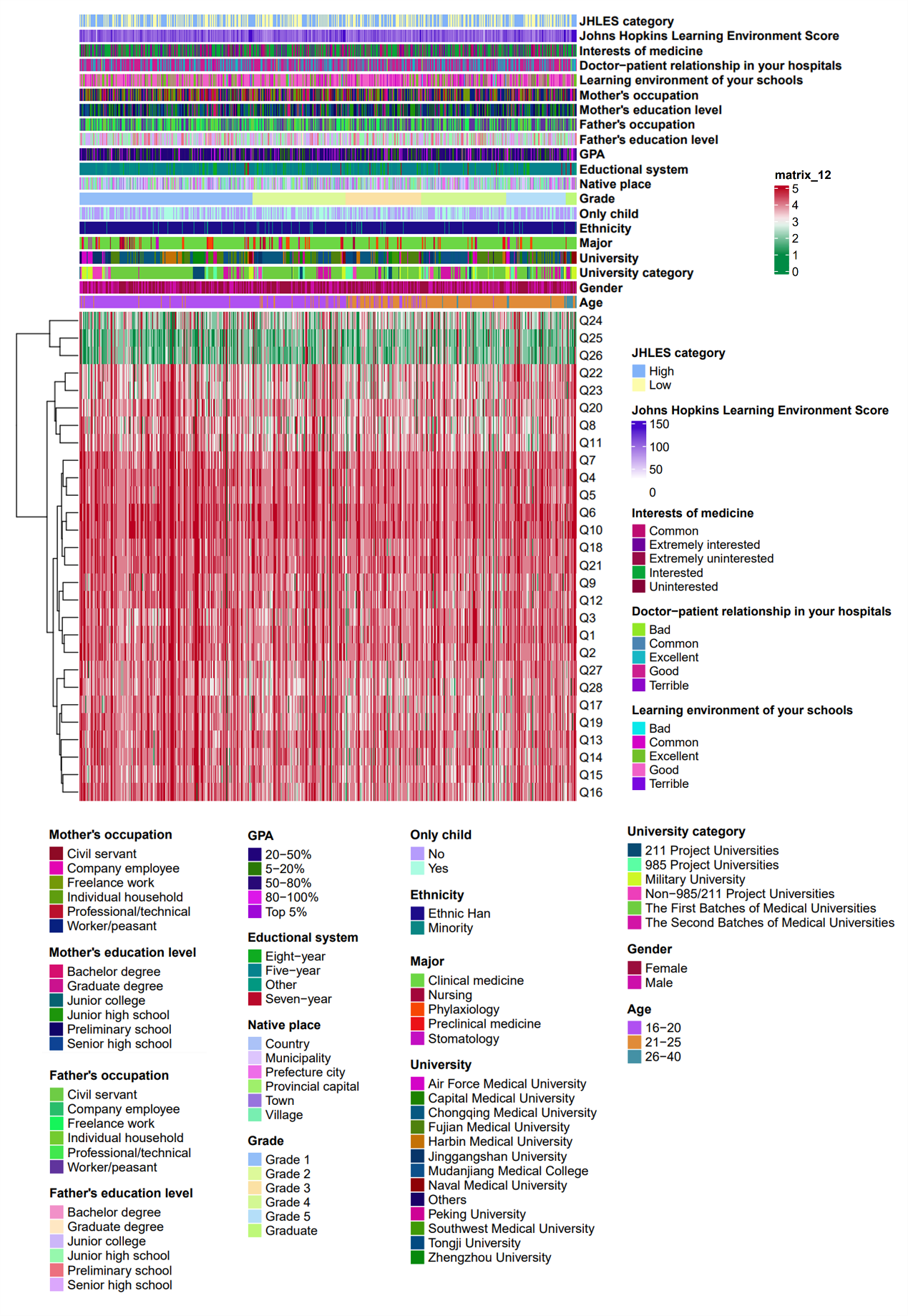


**Figure S1.** Heatmap: the distribution of 28 items’ scores of JHLES from students clustered by grade. JHLES, Johns Hopkins Learning Environment Scale; GPA, grade point average.


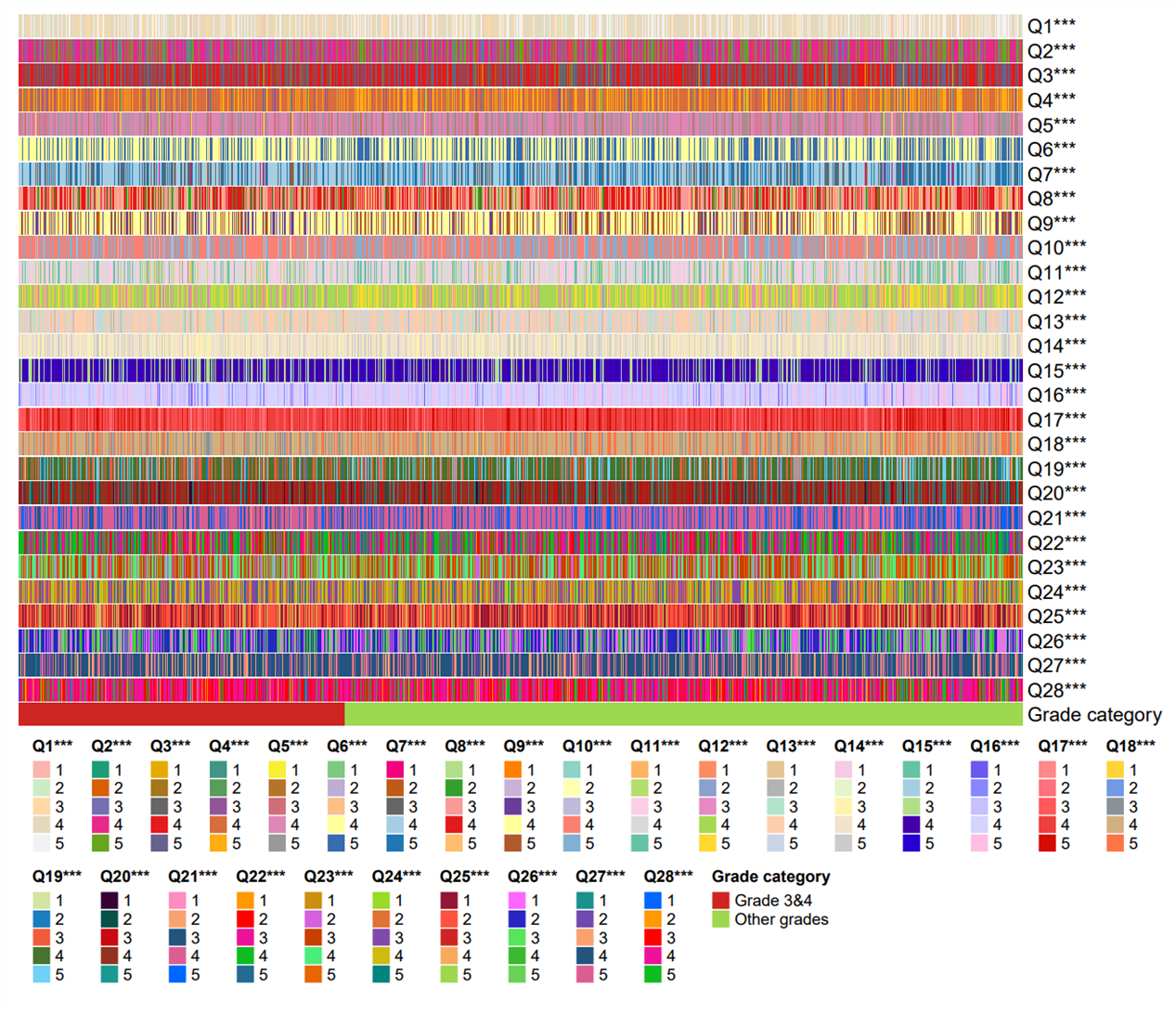


**Figure S2.** Pearson Chi-Square test identified that all 28 items of JHLES were significant different between grade 3 and 4 students and other graders.

**Table S1** | The detailed information and their structure coefficients for 28 items in JHLES.

| **28 items of 7 subscales in JHLES** | **Structure Coefficient** |
| --- | --- |
| **Community of peers** |  |
| 1. How connected do you feel to other SOM students? | 0.85 |
| 2. How supported do you feel in your personal and professional pursuits by other SOM students? | 0.77 |
| 3. It’s been easy to make friends at the SOM. | 0.73 |
| 4. I feel a sense of community at the SOM. | 0.67 |
| 5. To what extent have you felt a sense of belonging during your time as a student at the SOM? | 0.63 |
| 6. I’ve encountered an abundance of positive, inspiring role models among fellow students at the SOM. | 0.61 |
| **Faculty relationships** |  |
| 7. I feel that the SOM faculty I encounter are supportive of my professional goals. | 0.70 |
| 8. I feel that SOM faculty members have taken the time to get to know me. | 0.67 |
| 9. I feel that the SOM faculty I encounter genuinely care about my well-being. | 0.64 |
| 10. I’ve encountered an abundance of positive, inspiring faculty role models at the SOM. | 0.61 |
| 11. There are faculty members that I feel comfortable confiding in when important concerns come up. | 0.60 |
| 12. The faculty advisors in the Colleges Advisory Program are readily accessible and interested in students. | 0.54 |
| **Academic climate** |  |
| 13. Our medical school’s curriculum allows me to use my preferred learning style. | 0.75 |
| 14. I feel that course exams and assessments test my knowledge and abilities fairly. | 0.70 |
| 15. I understand the goals and objectives of the SOM curriculum. | 0.52 |
| 16. To what extent do you trust that the institution has fulfilled your needs as a medical student? | 0.51 |
| 17. The workload during medical school is manageable. | 0.53 |
| **Meaningful engagement** |  |
| 18. The SOM engages students as meaningful participants. | 0.69 |
| 19. The SOM is flexible and responsive to my needs as a student. | 0.61 |
| 20. I feel that I have a say in decision making about courses and curricular changes. | 0.60 |
| 21. The SOM encourages scholarship and innovation. | 0.51 |
| **Mentoring** |  |
| 22. I’ve found a mentor in a research field that interests me. | 0.74 |
| 23. I’ve found a mentor in a clinical specialty or discipline that I am passionate about. | 0.73 |
| **Inclusion and safety** |  |
| 24. I am concerned that students are mistreated at the SOM. | 0.72 |
| 25. I sense there is discrimination based on gender, race, ethnicity, or sexual identity at the SOM. | 0.47 |
| 26. I feel concerned at times for my personal safety at the SOM. | 0.46 |
| **Physical space** |  |
| 27.The preclinical SOM building has a significant effect on my perception of the learning environment. | 0.65 |
| 28.The work spaces where clinical teaching occurs contributes positively to my sense of the SOM learning environment. | 0.62 |

*JHLES, Johns Hopkins Learning Environment Scale; SOM, school of medicine.*
